# Morphology Characterization, Molecular Identification, and Pathogenicity of Fungal Pathogen Causing Kaffir Lime Leaf Blight in Northern Thailand

**DOI:** 10.3390/plants11030273

**Published:** 2022-01-20

**Authors:** Nakarin Suwannarach, Surapong Khuna, Jaturong Kumla, Ratchadawan Cheewangkoon, Piyawan Suttiprapan, Saisamorn Lumyong

**Affiliations:** 1 Research Center of Microbial Diversity and Sustainable Utilization, Chiang Mai University, Chiang Mai 50200, Thailand; Trio_9@hotmail.com (S.K.); Jaturong_yai@hotmail.com (J.K.); ratchadawan.c@cmu.ac.th (R.C.); piyawan.s@cmu.ac.th (P.S.); scboi009@gmail.com (S.L.); 2 Department of Biology, Faculty of Science, Chiang Mai University, Chiang Mai 50200, Thailand; 3 Department of Entomology and Plant Pathology, Faculty of Agriculture, Chiang Mai University, Chiang Mai 50200, Thailand; 4 Academy of Science, The Royal Society of Thailand, Bangkok 10300, Thailand

**Keywords:** citrus, fungal disease, *Lasiodiplodia chinensis*, leaf blight, topic area

## Abstract

Thailand is known to be the largest producer of kaffir lime leaf products in the global market. In 2021, leaf blight was found on kaffir lime plants (*Citrus hystrix* DC.) in Lamphun Province of northern Thailand. This disease has been associated with significant economic losses. However, there have been no prior reports of leaf blight on kaffir lime plants in Thailand or anywhere else in the world. In this study, causal fungi were isolated from lesions of kaffir lime plants and a total of three fungal isolates were obtained. All causal fungi were identified as *Lasiodiplodia chinensis* based on morphological characteristics and the phylogenetic analysis of combined sequences of the internal transcribed spacer (ITS) of ribosomal DNA, the translation elongation factor 1-alpha (*tef-1*), β-tubulin (*tub*), and RNA polymerase II subunit (*rbp2*) genes. Pathogenicity tests were conducted and the results revealed that all isolated fungi caused symptoms of leaf blight on inoculated leaves. This outcome was similar to symptoms that naturally occur and have been observed in the field. This is the first report on kaffir lime leaf blight caused by *L. chinensis*. Our study will provide information of high value for the development of effective strategies for the monitoring and prevention of this disease.

## 1. Introduction

Kaffir lime (*Citrus hystrix* DC.) is a citrus plant that is native to tropical Asia. This plant is commonly cultivated in tropical regions, especially Southeast Asia (Laos, Indonesia, Malaysia, Thailand, and Vietnam) [1,2,3]. The leaves and fruits of this plant are recognized as important ingredients in many traditional foods of Southeast Asia, particularly in Thai food [4,5]. In Thailand, kaffir lime is referred to as “Makrut lime” and “Thai lime”. Kaffir lime leaves and fruits have been beneficially used in traditional medicine to treat certain common ailments such as colds, congestion, and coughs [6,7,8]. They have also served as a digestive stimulant that can alleviate flatulence and indigestion, act as a blood purifier, and reduce high blood pressure [7,9]. Furthermore, the essential oils of kaffir lime leaves and fruits have been reported to display various bioactivities. They have also been acknowledged to exhibit antioxidant, antitussive, antileukemic, antihemorrhagic, antimicrobial, anticancer, anti-inflammatory, and antioxidative stress properties, while serving as functional components in skin-conditioning agents [4,10,11]. The essential oil of kaffir limes can also be used as a flavoring ingredient in the commercial food, perfumery, and cosmetic industries [12]. Presently, kaffir lime products, particularly those made from kaffir lime leaves, are marketed in fresh, frozen, and dried forms [6]. Thailand is known to be the largest producer of kaffir lime leaf products in the global market, followed by Indonesia, Malaysia, and India [5].

Global demand for kaffir lime products continues to rise in accordance with rapid population growth and the pursuit of healthier lifestyles. Consequently, plantation areas dedicated to the cultivation of kaffir lime plants have increased significantly. On the other hand, the incidence and severity of certain fungal-based diseases have also increased when plants have been cultivated in unsuitable locations [13,14,15]. Leaf blight caused by fungal pathogens is an important disease affecting citrus plants [16,17,18]. This disease is associated with yield losses in citrus cultivation, resulting in significant economic impacts [18,19,20]. In 2021, leaf blight caused by fungi was observed on kaffir limes collected from Lamphun Province in Thailand, with a degree of incidence within the range of 20 to 30%. Importantly, there had been no prior reports of leaf blight on kaffir lime plants. Therefore, the objective of this study was to isolate the causal fungal agents of this disease. The isolated fungi were identified and described using morphological and molecular data. Pathogenicity tests were then carried out and Koch’s postulates were applied to assess asymptomatic kaffir lime leaves using the isolated fungi.

## 2. Results

### 2.1. Sample Collection and Disease Symptoms

**Figure 1:**
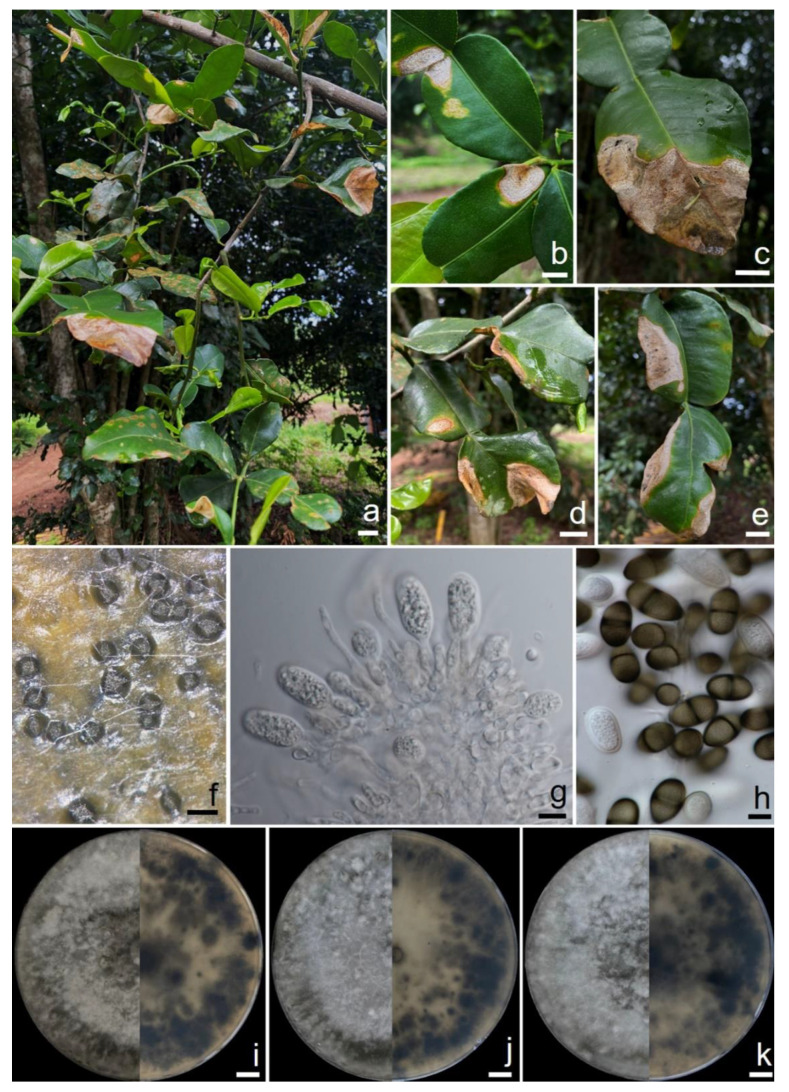
Natural symptoms of kaffir lime leaf blight caused by *Lasiodiplodia chinensis.* (**a**–**e**) Conidiomata on disease lesion. (**f**) Conidia developing on conidiogenous cells. (**g**) Conidia. (**h**) Colonies of *L. chinensis* CMU363 (**i**), CMU364 (**j**), and CMU365 (**k**) for three weeks on PDA (left, surface view and right, reverse view). Scale bars: a–e = 10 mm; f = 200 μm; g and h = 10 μm; i–k = 10 mm.

Samples of leaf blight of the kaffir lime plant (*C*. *hystrix*) were collected from one plantation area located in Lumphun Province, northern Thailand. Symptoms were characterized by the initial presence of small light-yellow spots (1.5 to 2 mm in diameter) with a yellow halo surrounding each lesion. These spots then expanded into irregular brown spots with dark-brown edges that were located at the margins and tips of the leaves. Lesions became enlarged and coalesced, causing the diseased leaves to appear blighted and desiccated. As a result of this disease, severely infected foliage turned brown, curled up, broke, shriveled, and died (Figure 1a–e). In humid environments, dark-brown to black conidiomata developed on the lesions and exuded spore masses that turned black after discharge (Figure 1f). The conidiomata were pycnidial, semi-immersed or sometimes superficial on the plant tissue, solitary, papillate, uniloculate, dark-brown to black, covered with dense brownish grey hyphal hairs, and 210–300 µm in diameter. Paraphyses were cylindrical, hyaline, smooth, thin-walled, initially aseptate, becoming up to 9-septate when mature and unbranched; the basal cells were occasionally swollen, up to 95 µm long and 3–7 µm wide. Conidiophores reduced to conidiogenous cells. Observed conidiogenous cells were holoblastic, hyaline, cylindrical to ampulliform, proliferating percurrently near apex, 8–18 × 4–7 µm (a mean value of 50 conidiogenous cells = 12.4 × 5.0 µm) (Figure 1g). Conidia were initially hyaline, unicellular, ovoid to ellipsoid, thick-walled with granular content, round at the apex, occasionally truncated at the base, and 18.5–25 × 12–14 µm (a mean value of 50 conidia = 22.0 × 12.7 µm, L/W ratio = 1.75, ranging from 1.43 to 2.08). They turned pale brown with a single median septum and longitudinal striations from the apex to base when mature (Figure 1h). Based on these morphological characteristics, the causal agent was initially identified as belonging to the genus *Lasiodiplodia*.

### 2.2. Fungal Isolation and Morphological Study

Pure cultures were isolated from a single conidial isolation. Three fungal isolates, CMU363, CMU364, and CMU365, which were of a similar morphology were obtained and deposited in the Culture Collection of Sustainable Development of Biological Resources Laboratory, Faculty of Science, Chiang Mai University (SDBR-CMU), Chiang Mai Province, Thailand, under the accession numbers SDBR-CMU363, SDBR-CMU364, and SDBR-CMU365, respectively. Fungal colonies on PDA were 85−90 mm in diameter and initially white with fluffy aerial mycelia. The fungal colonies then became pale olivaceous grey to olivaceous grey, while the reverse side became olivaceous grey to olivaceous black after three days of incubation at 30 °C (Figure 1i–k). Conidiomata, paraphyses, conidiophores, conidiogenous cells, and conidia were observed on PDA after two weeks of incubation at 30 °C. These characteristics matched the above-mentioned descriptions. Thus, all isolated fungi were initially identified as belonging to the genus *Lasiodiplodia*. Fungal identification was then further confirmed using multi-gene phylogenetic analyses.

### 2.3. Phylogenetic Results

Genomic DNA was extracted from three fungal cultures (SDBR-CMU363, SDBR-CMU364, and SDBR-CMU365) growing on PDA at 25 °C. The ITS, *tef-1*, *tub*, and *rpb2* sequences of each fungal isolate were deposited in the GenBank database (Table 1).

**Figure 2:**
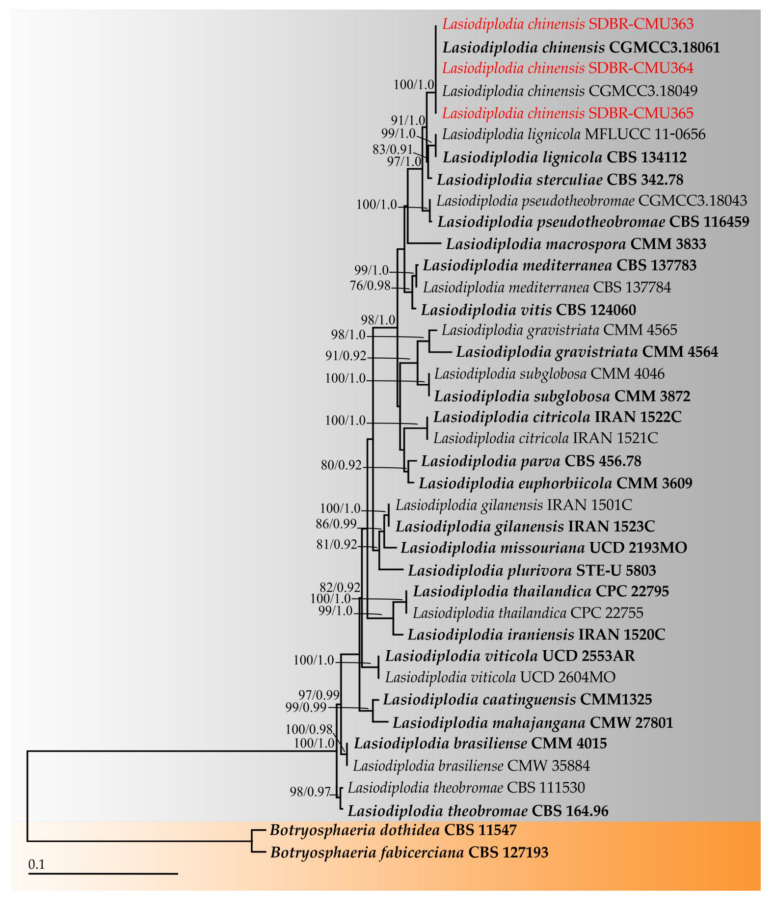
Phylogram derived from maximum likelihood analysis of 39 taxa of the combined ITS, *tef-1*, *tub,* and *rpb2* sequences. *Botryosphaeria fabicerciana* CBS 127193 and *B. dothidea* CBS 115476 were used as the outgroup. The numbers above branches represent bootstrap percentages (**left**) and Bayesian posterior probabilities (**right**). Bootstrap values ≥ 75% and Bayesian posterior probabilities ≥ 0.90 are shown. The scale bar represents the expected number of nucleotide substitutions per site. Sequences of fungal species obtained in this study are in red. Type species are in bold.

The combined ITS, *tef-1*, *tub,* and *rpb2* sequence dataset consisted of 39 taxa and the aligned dataset was comprised of 1839 characters including gaps (ITS: 1–557; *tef-1*: 558–889; *tub*: 890–1307; and *rpb2*: 1308–1839). ML analysis of the combined dataset yielded a best scoring tree with a final ML optimization likelihood value of −5129.6382. The matrix contained 347 distinct alignment patterns with 18.22% undetermined characters or gaps. Estimated base frequencies were recorded as follows: A = 0.2215, C = 0.2871, G = 0.2649, T = 0.2264; substitution rates AC = 0.9588, AG = 3.3197, AT = 1.3392, CG = 1.0545, CT = 7.3927, GT = 1.0000; and gamma distribution shape parameter alpha = 0.6280. The gamma distribution shape parameter alpha value was equal to 0.1724 and the Tree-Length value was equal to 0.4173. In addition, the final average standard deviation of split frequencies at the end of the total MCMC generations was calculated as 0.00825 through BI analysis. Phylograms of the ML and BI analyses were similar in terms of topology (data not shown). The phylogram obtained from the ML analysis presented in Figure 2 was constructed concordantly with support from previous studies [24,42,43,44]. The phylogram successfully assigned the three fungal isolates obtained in this study into the same clade of *L*. *chinensis* containing the type species (CGMCC3.18061). This clade formed a monophyletic clade with high BS (100%) and PP (1.0) supports. *Lasiodiplodia chinensis* formed a sister taxon with *L*. *lignicola* and *L*. *sterculiae*, exhibiting high statistical support (91% BS and 1.0 PP). Therefore, the three fungal isolates obtained in this study were identified as *L*. *chinensis* based on their morphological characteristics and multi-gene phylogenetic analyses.

### 2.4. Pathogenicity Test

**Figure 3:**
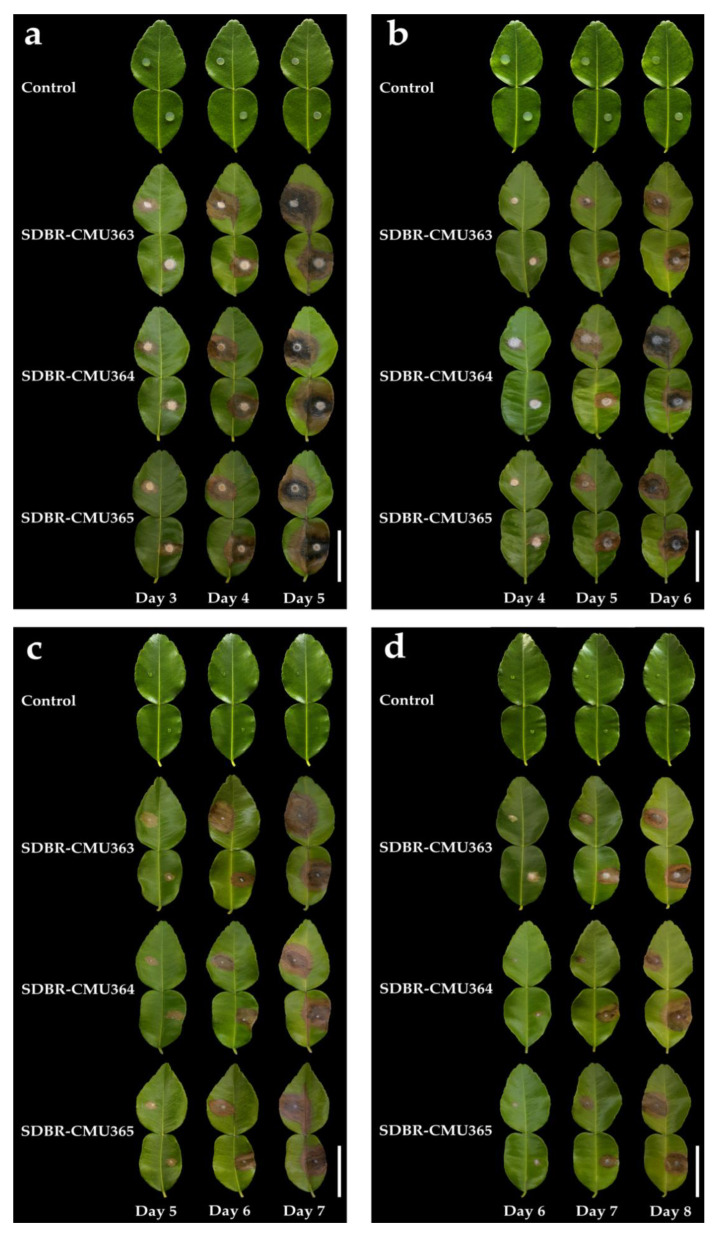
Pathogenicity test using *L. chinensis* SDBR-CMU363, SDBR-CMU364, and SDBR-CMU365 on kaffir lime leaves after inoculation by mycelial plug (**a**,**b**) and conidial suspension (**c**,**d**). The experiments of wounded (**a**,**c**) and unwounded (**b**,**d**) leaves. Scale bars = 50 mm.

The mycelial plug and conidia from all fungal isolates were used in this experiment. Initial symptoms were observed on wounded and unwounded leaves at three and four days, respectively, after inoculation by mycelial plug. Initially, small light-brown to brown spots appeared on the leaves. The lesions then enlarged rapidly and became brown to dark-brown spots that were covered with sparse white mycelia. The diameters of the lesions on the wounded and unwounded leaves were within the ranges of 2.0–3.1 and 1.7–2.5 cm after four and six days of incubation, respectively (Figure 3a,b). The lesions would then spread through entire leaves and coalesce within seven and nine days after the occurrence of necrosis. After that, the leaves became blighted and desiccated. Additionally, initial disease symptoms of the wounded and unwounded leaves inoculated with conidial suspensions were observed three and four days after incubation. Symptoms observed on the wounded and unwounded leaves were circular brown to dark-brown spots 1.5–2.5 and 1.2–2.0 cm in diameter after seven and eight days of incubation, respectively (Figure 3c,d). Lesions then covered entire leaves and coalesced within ten days. These disease symptoms were similar to those seen on the leaves inoculated with mycelial plugs. However, plant disease symptoms were not observed in any inoculation treatments involving PDA plugs and sterile distilled water for both wounded and unwounded leaves. Koch’s postulates were fulfilled by the fungi re-isolated from symptomatic leaf tissue and then grown on PDA. The re-isolated fungi were identified as *L. chinensis*.

## 3. Discussion

Many diseases caused by fungi, bacteria, and viruses can affect the leaves, stems, roots, and fruits of citrus plants, from seedlings to mature stages [45,46,47,48,49]. In this study, three isolates of *L. chinensis* were isolated from the lesions of leaf blight on kaffir lime plants collected from northern Thailand. All isolated fungi were identified by their morphological and molecular characteristics according to the identification approaches employed in previous studies [24,42,43,44]. To fulfill Koch’s postulates, pathogenicity was tested for all strains that had developed the same symptoms as those observed in the field. Our findings are supported by those of previous studies which reported that *Lasiodiplodia* is an economically important plant pathogen and that the *Lasiodiplodia* species have been reported to cause various disease symptoms in citrus plants in tropical and subtropical regions throughout the world [50,51,52,53]. For examples, *L. brasiliense*, *L. citricola*, *L. iraniensis*, *L. pseudotheobromae*, *L. theobromae,* and *L. subglobosa* were found to cause necrotic lesions and gummosis on Persian lime plants (*C. latifolia*) in several regions of Mexico [54]. In Pakistan, *L. iraniensis* and *L. pseudotheobromae* have been identified as the causal agents for tip dieback in *C. reticula* and trunk cankers in *C. reticulate*, respectively [55,56]. Moreover, previous studies have reported that *L. citricola*, *L. guilinensis*, *L. huangyanensis*, *L. iraniensis*, *L. linhaiensis*, *L. microconidia*, *L. ponkanicola*, *L. pseudotheobromae*, and *L. theobromae* caused branch diseases in citrus plants in China [43,57,58]. Furthermore, bot gummosis in citrus plants, caused by *L*. *pava* and *L*. *theobromae*, has been reported in the USA [59] and Chile [60], respectively. There have been no reports of leaf blight disease caused by *Lasiodiplodia* species in citrus plants. However, leaf blight disease in citrus plants caused by *Fusarium solani* and *Colletotrichum gloeosporioides* has been reported in the USA [17] and India [16], respectively.

In Thailand, *Lasiodiplodia* species have been the known cause of many plant diseases prior to this research. For examples, *L. theobromae* was found to be a causal agent of spadix rot in flamingo lily plants (*Anthurium andraeanum*) [61] and fruit rot on certain melon species (*Cucumis melo*) [62]. Fruit rot in postharvest longan (*Dimocarpus longan*) fruits [63], stem rot disease on durian trees (*Durio zibethinus*) [64], and leaf spots on *Cynometra malaccensis* [65] have been reported to be caused by *L. pseudotheobromae*. *Lasiodiplodia pseudotheobromae* and *L. viticola* have been reported to cause fruit rot and stem-ends in mango plants (*Mangifera indica*) [66]. However, there have been no prior reports of leaf blight on kaffir lime crops in Thailand or anywhere else in the world. Thus, we propose that leaf blight disease caused by *L. chinensis* should be recognized as a new disease affecting the kaffir lime plant. *Lasiodiplodia chinensis* has been reported and classified as a saprobic or pathogenic fungus associated with the bog blueberry plant (*Vaccinium uliginosum*), canarium nut tree (*Canarium parvum*), Malva nut tree (*Sterculia lychnophora*), rose myrtle plant (*Rhodomyrtus tomentosa*), and rubber trees (*Hevea brasiliensis*) in China [24].

It can be difficult to evaluate the harm caused by *L. chinensis* to kaffir lime plants during the cultivation period. The fungus can infect kaffir lime leaves in the field; however, due to the wide host range associated with *L. chinensis*, infection can possibly come from other plants in the surrounding area. At the same time, since this fungus has been found to be saprobic or pathogenic in several tropical and subtropical trees, it can produce pycnidia and release conidia that then accumulate in the atmosphere surrounding the plants as well as in the soil [24]. Follow-up studies are needed to clarify the inoculum source of the disease and the meteorological conditions that impact infection and disease development. Additionally, the distribution of this disease in other regions of Thailand should also be investigated.

## 4. Materials and Methods

### 4.1. Sample Collection

Leaf blight of the kaffir lime plant (*Citrus hystrix*) was collected from a plantation area in Lumphun Province (18°32′02″ N 99°07′30″ E, elevation 382 m), northern Thailand, in 2021. Twenty symptomatic leaves were randomly collected from this plantation. Leaf samples were kept in sterile zip-lock plastic bags and carried to the laboratory within 48 h of collection. After being transferred to the laboratory, symptomatic leaves were examined using a stereo microscope (Nikon H55OS, Tokyo, Japan) and kept in a plastic box with wet filter paper to induce sporulation. The fungal structures (such as conidiomata, conidiophore, conidiogenous cells, and conidia) were examined under a light microscope (Nikon Eclipse Ni-U, Tokyo, Japan). Assessments were based on at least 50 measurements of each structure using the Tarosoft (R) Image Frame Work program.

### 4.2. Fungal Isolation and Morphological Study

Leaf samples were processed for the isolation of fungal causal agents. The causal fungi were isolated from lesions using a single conidial isolation on 1.0% water agar containing 0.5 mg/l streptomycin under a stereo microscope according to the method described by Choi et al. [67]. The isolated plates were incubated at 25 °C for 24–48 h, and the germinated conidia were transferred onto potato dextrose agar (PDA; Conda, Madrid, Spain) containing 0.5 mg/l streptomycin. Pure fungal isolates were deposited in the Culture Collection of SDBR-CMU Laboratory, as previously mentioned.

### 4.3. Molecular Study

#### 4.3.1. DNA Extraction, PCR Amplification, and Sequencing

Genomic DNA was extracted from the fungal cultures of each isolate that grew on PDA at 25 °C for five days using a Fungal DNA Extraction Kit (FAVORGEN, Ping-Tung, Taiwan) according to the manufacturer’s protocol. The ITS, *tef-1*, *tub,* and *rbp2* genes were amplified by polymerase chain reaction (PCR) using ITS4/ITS5 primers [68], EF1-983F/EF1-2218R primers [69], Bt2a/Bt2b primers [70], and RPB2-LasF/RPB2-LasR primers [22], respectively (Table 2). The amplification program for all four genes was performed in separate PCR reactions and consisted of an initial denaturation step at 95 °C for 5 min followed by 35 cycles of denaturation at 95 °C for 30 s, an annealing step at 52 °C for 45 s (ITS), 55 °C for 1 min (*tub* and *rbp2*) and 56 °C for 1 min (*tef-1*), and an extension step at 72 °C for 1 min on a peqSTAR thermal cycler (PEQLAB Ltd., Fareham, UK). PCR products were checked using gel electrophoresis and were purified using a PCR clean up Gel Extraction NucleoSpin^®^ Gel and PCR Clean-up Kit (Macherey-Nagel, Düren, Germany) according to the manufacturers’ protocols. Purified PCR products were directly sequenced. The sequences were automatically determined in a genetic analyzer at the 1^ST^ Base Company (Kembangan, Malaysia) using the PCR primers mentioned above.

#### 4.3.2. Sequence Alignment and Phylogenetic Analyses

The analysis of the ITS, *tef-1*, *tub,* and *rpb2* sequences was conducted with the use of similarity searches employing the BLAST program available at NCBI (http://blast.ddbj.nig.ac.jp/top-e.html, accessed on 11 November 2021). The sequences from this study and those obtained from previous studies together with sequences downloaded from the nucleotide GenBank database are listed in Table 1. Multiple sequence alignment was performed with MUSCLE [71] and improved where necessary using BioEdit v. 6.0.7 [72]. Phylogenetic analysis was carried out based on the combined dataset of ITS, *tef-1*, *tub,* and *rpb2*. *Botryosphaeria fabicerciana* CBS 127193 and *B*. *dothidea* CBS 115476 were used as the outgroup. A phylogenetic tree was constructed using maximum likelihood (ML) and Bayesian inference (BI) methods. ML analysis was carried out on RAxML v7.0.3 under the GTRCAT model with 25 categories and 1000 bootstrap (BS) replications [73,74] via the online portal CIPRES Science Gateway v. 3.3 [75]. BI analysis was performed with MrBayes v3.2.6 [76]. For the BI analysis, six simultaneous Markov chains were run for one million generations with random initial trees, wherein every 1000 generations were sampled. A burn-in phase was employed to discard the first 2000 of the trees, while the remaining trees were used to construct the 50% majority-rule consensus phylogram with calculated Bayesian posterior probabilities (PP). Tree topologies were visualized in FigTree v1.4.0 [77].

### 4.4. Pathogenicity Tests

Asymptomatic leaves were collected from kaffir lime plants cultured in a disease-free area of Chiang Mai Province, Thailand, and kept in sterile plastic bags. The leaves were carried to the SDBR-CMU laboratory within 2 h of being collected. Leaf samples were processed immediately in terms of their pathogenicity after reaching the laboratory. Leaves were surface disinfected using 0.1% (*v*/*v*) sodium hypochlorite for 3 min and then washed three times with sterile distilled water. The surface disinfected leaves were then air-dried under laminar flow for 10 min. After being air-dried, a uniform wound (5 pores, 3 mm in width) was made at the equator of each leaf using aseptic needles. Fungal mycelia and conidia were used as inocula. Mycelial plugs (5 mm in diameter) of each fungal isolate cut off from the margin of the colonies grown on PDA at 25 °C for five days were transferred onto wounded and unwounded leaves. Plugs of PDA were used as controls. Conidial suspensions were collected from each fungal culture grown on PDA at 25 °C for three weeks and suspended in sterile distilled water. The suspension was filtered through two layers of sterile cheesecloth, diluted in distilled water with 0.05% (*v*/*v*) Tween 20, and adjusted to 1 × 10^6^ conidia/mL using a hemacytometer. Ten microliters of the conidial suspension were dropped onto the wounded and unwounded leaves. Subsequently, control leaves were dropped with sterile distilled water. The inoculated leaves were arranged (5 leaves per box) in 4 L plastic boxes at conditions of 90% relative humidity. The plastic boxes were stored in a growth chamber at 25 °C under a 12-h period of light for one week. Ten replications were performed for each treatment. The experiments were independently repeated twice. To authenticate the causal agent, fungi were re-isolated from the lesions according to the method described by Suwannarach et al. [78].

## 5. Conclusions

Leaf blight on kaffir lime plants caused by *L. chinensis* was found in northern Thailand in 2021. The fungus was isolated and identified based on morphological characteristics and multi-gene phylogenetic analyses. The pathogenicity of the disease was determined using fungal mycelia and conidia, which had developed the same symptoms under artificial inoculation conditions as those observed in the field. This is the first report of kaffir lime leaf blight caused by *L*. *chinensis*. Consequently, further studies involving the distribution and control of this disease will need to be conducted. In order to address the significant economic losses associated with this disease, the development of effective strategies for its monitoring and prevention will be critical in the future.

## Figures and Tables

**Table 1:** Details of sequences used for phylogenetic analysis.

Fungal Taxa	Strain/Isolate	GenBank Accession Number				Reference
		ITS	*tef-1*	*tub*	*rpb2*	
*Lasiodiplodia brasiliense*	CMM 4015 ^T^	JX464063	JX464049	−	−	[21]
*L. brasiliense*	CMW 35884	KU887094	KU886972	KU887466	KU696345	[22]
*L. caatinguensis*	CMM1325 ^T^	KT154760	KT008006	KT154767	−	[23]
*L. chinensis*	CGMCC3.18061 ^T^	KX499889	KX499927	KX500002	KX499965	[24]
*L. chinensis*	CGMCC3.18049	KX499878	KX499916	KX499991	KX499954	[24]
*L. chinensis*	SDBR-CMU363	OL989102	OL989839	OL989842	OL989845	This study
*L. chinensis*	SDBR-CMU364	OL989137	OL989840	OL989843	OL989846	This study
*L. chinensis*	SDBR-CMU365	OL989141	OL989841	OL989844	OL989847	This study
*L. citricola*	IRAN 1522C ^T^	GU945354	GU945340	KU887505	KU696351	[22,25]
*L. citricola*	IRAN 1521C	GU945353	GU945339	KU887504	KU696350	[22,25]
*L. euphorbiicola*	CMM 3609 ^T^	KF234543	KF226689	KF254926	−	[26]
*L. gilanensis*	IRAN 1523C ^T^	GU945351	GU945342	KU887511	KU696357	[22,25]
*L. gilanensis*	IRAN 1501C	GU945352	GU945341	KU887510	KU696356	[22,25]
*L. gravistriata*	CMM 4564 ^T^	KT250949	KT250950	−	−	[27]
*L. gravistriata*	CMM 4565	KT250947	KT266812	−	−	[27]
*L. iraniensis*	IRAN 1520C ^T^	GU945348	GU945336	KU887516	KU696363	[22,25]
*L. lignicola*	CBS 134112 ^T^	JX646797	KU887003	JX646845	KU696364	[22,28]
*L. lignicola*	MFLUCC 11-0656	JX646798	−	JX646846	−	[28]
*L. macrospora*	CMM 3833 ^T^	KF234557	KF226718	KF254941	−	[26]
*L. mahajangana*	CMW 27801 ^T^	FJ900595	FJ900641	FJ900630	KU696365	[29]
*L. mediterranea*	CBS 137783 ^T^	KJ638312	KJ638331	KU887521	KU696368	[22,30]
*L. mediterranea*	CBS 137784	KJ638311	KJ638330	KU887522	KU696369	[22,30]
*L. missouriana*	UCD 2193MO ^T^	HQ288225	HQ288267	HQ288304	KU696370	[22,31]
*L. parva*	CBS 456.78 ^T^	EF622083	EF622063	KU887523	KU696372	[22,32]
*L. plurivora*	STE-U 5803 ^T^	EF445362	EF445395	KU887524	KU696374	[22,33]
*L. pseudotheobromae*	CBS 116459 ^T^	EF622077	EF622057	EU673111	KU696376	[22,32]
*L. pseudotheobromae*	CGMCC3.18043	KX499872	KX499910	KX499985	KX499948	[24]
*L. sterculiae*	CBS 342.78 ^T^	KX464140	KX464634	KX464908	KX463989	[34]
*L. subglobosa*	CMM 3872 ^T^	KF234558	KF226721	KF254942	−	[26]
*L. subglobosa*	CMM 4046	KF234560	KF226723	KF254944	−	[26]
*L. thailandica*	CPC 22795 ^T^	KJ193637	KJ193681	−	−	[35]
*L. thailandica*	CPC 22755	KM006433	KM006464	−	−	[36]
*L. theobromae*	CBS 164.96 ^T^	AY640255	AY640258	KU887532	KU696383	[22,37]
*L. theobromae*	CBS 111530	EF622074	EF622054	KU887531	KU696382	[22,32]
*L. viticola*	UCD 2553AR ^T^	HQ288227	HQ288269	HQ288306	KU696385	[22,31]
*L. viticola*	UCD 2604MO	HQ288228	HQ288270	HQ288307	KU696386	[22,31]
*L. vitis*	CBS 124060 ^T^	KX464148	KX464642	KX464917	KX463994	[34]
*Botryosphaeria dothidea*	CBS 115476 ^T^	AY236949	AY236898	AY236927	EU339577	[38]
*B. fabicerciana*	CBS 127193 ^T^	HQ332197	HQ332213	KF779068	MF410137	[39,40,41]

Superscript “T” represents type species. “−” represents the absence of sequence data in GenBank.

**Table 2:** Details of primers and the obtained PCR products in this study.

Gene	Primer Name	Primer Sequence	The Obtained Length (bp)		
			SDBR-CMU363	SDBR-CMU364	SDBR-CMU365
ITS	ITS4	5′-TCCTCCGCTTATTGATATGC-3′	540	522	529
	ITS5	5′-GGAAGTAAAAGTCGTAACAAGG-3′			
*tef-1*	EF1-983F	5′-GCYCCYGGHCAYCGTGAYTTYAT-3′	955	943	932
	EF1-2218R	5′-ATGACACCRACRGCRACRGTYTG-3′			
*tub*	Bt2a	5′-GGTAACCAAATCGGTGCTGCTTTC-3′	890	810	850
	Bt2b	5′-ACCCTCAGTGTAGTGACCCTTGGC-3′			
*rbp2*	RPB2-LasF	5′-GGTAGCGACGTCACTCCT-3′	593	580	591
	RPB2-LasR	5′-GCGCAAATACCCAGAATCAT-3′			

## Data Availability

The DNA sequence data obtained from this study have been deposited in GenBank under accession numbers ITS (OL989102, OL989137, OL989141), *tef-1* (OL989839, OL989840, OL989841), *tub* (OL989842, OL989843, OL989844), and *rpb2* (OL989845, OL989846, OL989847).
